# Supplementary material for: The Nucleoid-Associated Protein Fis Represses Type 3 Fimbriae to Modulate Biofilm and Adherence Formation in Klebsiella pneumoniae
Source: Microorganisms. 2025 Nov 13;13(11):2591. doi: 10.3390/microorganisms13112591 (PMC12655184; doi:10.3390/microorganisms13112591)
Supplement: Supplementary file 1 [file microorganisms-13-02591-s001.zip › microorganisms-3961947-supplementary.pdf]

Table S1

Primers used in this study*. Sequences are shown in 5’ to 3’ direction.

For qPCR	Target gene	RE
mrkA-F: CCATGCAGCTGATACCAATG	*mrkA*	
mrkA-R: GCAGCCTGGCAGTTAGAGAC		
mrkH-F: AAAATCAAACGCCTCACGAC	*mrkH*	
mrkH-R: TGCGATGGGTCTGAATATGA		
rpoD-F: GATCTGATCACCGGTTTCGT	*rpoD*	
rpoD-R: CTGTTGTCGTCATCGCTGTT		
For mutagenesis		
H1P1- Fis: AACACATTGTAAGGATAACTTATGAACAAGACTCAA<br>CTGATTGATTGTAGGCTGGAGCTGCTTCG	*fis*	
H2P2-Fis: CAAACACTTAACTGATTAGTATCAGTTCATGCCG<br>TATTTTTTCAG CATATGAATATCCTCCTTAG		
For characterization of the mutant		
Kpn-Fis-F: AGGCATACTTCGAAAATTTTTGC	*fis*	
Kpn-Fis-R: TAATGAGTTCTGTTGGCGCAG		
For *cis*-complementation		
Fis-BamHI-F: CTT***GGATCC***ACGTCTCCTGGTATCTTCAGG	*fis*	*Bam*HI
Fis-XhoI-R: GAA***CTCGAG***CAACGCTTCGATCTTTATCAG		*Xho*I
Fis-H3P3-R: CAAGCAAGCAACTGACTAGTATCAGTTCATGCC<br>GTATTTTTTCAG TAATACGACTCACTATAGGGC	*fis*	
To clone *fis* into the expression vector pMPMT6		
Fis-F-NcoI-Kpn: GAA***CCATGG***TCGAACAACGCGTAAATTCTGAC	*fis*	*Nco*I
Fis-R-Hind6H-Kpn: TGA***AAGCTT***TCAATGATGATGATGATG<br>ATGGTTCATGCCGTATTTTTTCAG	*fis*	*Hind*III
For EMSA test probes		
mrkA-F: ATGGCGGTTTGATGGCGTAAAC	*mrkA*	
mrkA-R: TGCTGCAGAGAGAAGAACCTTTTTC		
mrkH-F: AGGCGCAGGAGTTGAACGAGGTC	*mrkH*	
mrkH-R: GGTCTTTATCGTTCCCTCTGTCATATG		
fbpA-F: TTCCTGACCAGCGAGCTGCCG	*fbpA*	
fbpA-R: CCCCAGTACTCCCAGCTGTGC		
For cloning in pKK232-9		
mrkA-BamF-pKK: GGGGGATCCATGGGCTGCCCTTGTTCAG		
mrkA-HindR-pKK: GGGAAGCTTTGCCATTTCCTTGTCAGAGTGA		
mrkH-BamF-pKK: GGGGGATCCGCTGATCCCCGGCACCTC		
mrkH-HindR-pKK: GGGAAGCTTCCCTTGTAAATAGTTGTCGTGAGG		

*The sequence corresponding to the template plasmid pKD4 is underlined. Italic/bold letters indicate the respective restriction enzyme site in the primer. RE: Restriction Enzyme.
